# Hernia of the cauda equina nerve caused by occult cerebrospinal fluid leakage post lumbar spinal stenosis operation: A case report

**DOI:** 10.1097/MD.0000000000044098

**Published:** 2025-08-29

**Authors:** Chun Zhang, Jiaxin Fu, Shuzhang Guo, Zhi Liu

**Affiliations:** a Department of Orthopedics, The Third Central Hospital of Tianjin, Tianjin, China.

**Keywords:** cauda equina nerve compression, lumbar spine postoperatively, occult cerebrospinal fluid leak

## Abstract

**Rationale:**

Occult cerebrospinal fluid (CSF) leakage after lumbar spine surgery is common; however, cases in which CSF leakage leads to cauda equina tethering are rare and may result in severe neurological symptoms. This study elucidates the diagnostic challenges and management strategies for this rare complication through a representative case report.

**Patient concerns:**

A 74-year-old man was diagnosed with lumbar spinal stenosis and lumbar disc herniation. He underwent lumbar decompression, fusion, and internal fixation. On the day following discharge, he developed bilateral hip and lower limb pain and numbness that did not respond to oral analgesics.

**Diagnoses:**

Tethered cauda equina syndrome caused by occult CSF leakage and a dural defect following lumbar spinal stenosis surgery.

**Interventions:**

A second surgical exploration revealed an extradural pseudocyst and a dural defect. Clear CSF was observed within the cyst, and the cauda equina was entrapped and tethered. After careful release and dural repair, the patient’s symptoms improved significantly.

**Outcomes:**

The patient experienced pain relief and regained independent mobility and daily function.

**Lessons:**

Prolonged negative pressure drainage in the presence of CSF leakage may lead to nerve root tethering. Drainage duration should be carefully balanced to prevent infection while avoiding nerve entrapment. Timely surgical intervention can effectively relieve nerve compression and improve outcomes. This case highlights the need for heightened vigilance regarding delayed neurological deterioration following spinal surgery, emphasizing the critical importance of early recognition and intervention.

## 1. Introduction

Cerebrospinal fluid (CSF) leakage is a common complication following posterior decompressive laminectomy with nucleus pulposus removal for lumbar spinal stenosis.^[1]^ The incidence of dural damage during spinal surgery is 0.6% to 17.4%, and the incidence of postoperative CSF leakage is 1% to 17%.^[1,2]^ Due to hypertrophy of the ligamentum flavum, associated with the degenerative lumbar spinal stenosis, there is close adhesion between the ligamentum flavum and dura mater.^[3]^ During decompression and removal of the ligamentum flavum or decompression at the edge of the lamina, the dura mater is susceptible to inadvertent puncture, resulting in CSF leakage.^[4]^ For cases of minimal and hidden dural defects, specific treatment is often unnecessary.^[4]^ In this study, we report a case of hidden CSF leakage after surgery for lumbar spinal stenosis, which led to cauda equina nerve traction and neurological symptoms in the lower limbs. The patient’s condition improved after prompt surgical exploration and treatment. The diagnosis, clinical presentation, and therapeutic outcome are reported.

## 2. Case report

### 2.1. Patient information and initial presentation

A 74-year-old retired male worker was admitted on May 10, 2022, with a chief complaint of low back pain accompanied by radiating pain in the left lower limb for over 1 month. The patient had no history of significant trauma. Symptoms began in mid-to-late March 2022, initially presenting as persistent dull low back pain that worsened with physical activity and was not relieved by rest. He also experienced numbness in the left great toe. While the symptoms were initially alleviated somewhat by rest, they gradually progressed and became aggravated by coughing and sneezing.

On admission, the physiological lumbar lordosis was absent. Tenderness was noted over the left side of the L5 spinous process and the left paraspinal region at the L4/5 vertebral level, accompanied by radiating pain in the left lower limb and numbness of the left great toe. Lumbar range of motion was restricted: flexion 30°, extension 30°, and 30° of rotation bilaterally. Pain sensation was markedly diminished over the skin of the left great toe. Muscle strength of the quadriceps, hamstrings, semitendinosus, semimembranosus, tibialis anterior, tibialis posterior, extensor hallucis longus, and peroneus longus was normal (grade V) in both lower limbs. The left patellar and Achilles tendon reflexes were diminished (+), while reflexes on the right side were normal (++). Babinski sign was negative bilaterally, and no clonus was observed at the patella or ankle. The straight leg raise test and its reinforcement test were positive on the left side (+) and negative on the right (−). Muscle tone was normal in both lower limbs, and the dorsalis pedis and posterior tibial artery pulses were palpable and normal bilaterally.

**Figure 1.**
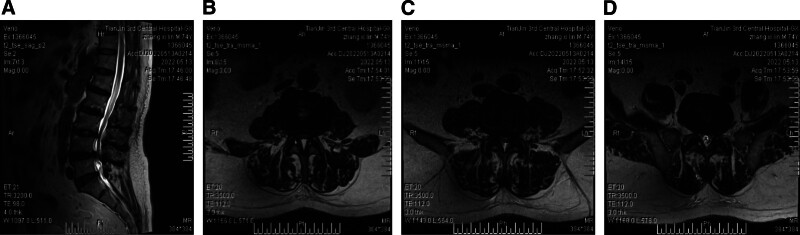
Preoperative lumbar spine MRI findings. (A) Sagittal plane showing lumbar disc herniation at L3/4, L4/5, and L5/S1 levels. (B) Axial plane at L3/4 level demonstrating thickening of the ligamentum flavum and spinal canal stenosis. (C) Axial plane at L4/5 level demonstrating thickening of the ligamentum flavum and spinal canal stenosis. (D) Axial plane at L5/S1 level demonstrating thickening of the ligamentum flavum and spinal canal stenosis. MRI = magnetic resonance imaging.

Lumbar magnetic resonance imaging (MRI) revealed ligamentum flavum hypertrophy from L3/4 to L5/S1, spinal canal stenosis, and bilateral foraminal narrowing with significant thecal sac compression (Fig. 1A–D). The findings were consistent with lumbar spinal stenosis and disc herniation.

### 2.2. Initial surgery and postoperative course

On May 16, the patient underwent L3–L5 posterior lumbar decompression, discectomy, interbody fusion, and pedicle screw fixation under combined general anesthesia. Intraoperatively, marked spinal canal stenosis and significant ligamentum flavum hypertrophy were observed, with notable compression of the L4/5 thecal sac. A small dural tear was suspected due to a minor amount of clear CSF leakage; however, no definitive defect was identified and no repair was performed. Estimated blood loss was approximately 500 mL, with 375 mL autologous transfusion administered. Postoperatively, the patient received anti-infective therapy, analgesia, neurotrophic support, and fluid replacement. On postoperative day 2, numbness in the left foot improved significantly, but the patient reported discomfort in the sacroiliac region. Physical examination revealed decreased pain sensation over the bilateral anterior and posterior thighs, calves, dorsum and plantar surfaces of the feet, and the saddle area. Muscle strength in both lower limbs was grade V with normal muscle tone. Straight leg raising and enhanced tests were negative bilaterally. Patellar reflexes were brisk (++), Achilles reflexes absent, and Babinski signs negative. Bilateral pitting edema was present in the lower limbs. Surgical site drainage measured 740 mL of dark red turbid fluid, raising suspicion of CSF leakage. Cefuroxime was initiated and switched to vancomycin on postoperative day 3. Due to increased drainage volume, the drainage tube was left in place for an extended period and removed on postoperative day 10 after continuous drainage. Sutures were removed on postoperative day 15, after which the patient ambulated with a brace without significant pain or discomfort. Neurological examination remained unchanged, the surgical incision healed well, and clinical improvement was noted. The patient was discharged on June 1.

### 2.3. Recurrence and imaging evaluation

**Figure 2.**
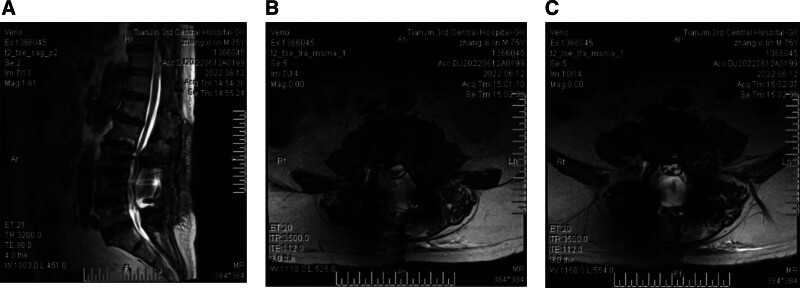
Postoperative MRI showing extradural pseudocyst formation and signs of cauda equina traction. (A) Sagittal T2-weighted image demonstrates a large, well-defined hyperintense lesion posterior to the thecal sac spanning L3 to L5 levels, consistent with an extradural pseudocyst. (B, C) Axial T2-weighted images at the L3 and L5 levels show the pseudocyst occupying the dorsal spinal canal, compressing the thecal sac anteriorly. The cauda equina nerve roots appear clustered, suspended, and disorganized, indicating traction-related changes. MRI = magnetic resonance imaging.

On June 2, the patient experienced sudden onset of severe bilateral buttock pain that was persistent and unrelieved, accompanied by significant numbness in both lower limbs. Bed rest and oral analgesics were ineffective. The patient was readmitted on June 8. Physical examination revealed hypesthesia in both lower limbs and perineal region, with muscle strength graded as V and normal muscle tone. Bilateral straight leg raising and enhanced tests were negative. Patellar reflexes were brisk (++), Achilles reflexes were absent (−), and Babinski signs were negative. Bilateral pitting edema was present in the lower limbs, and the dorsalis pedis and posterior tibial arterial pulses were weakened. Follow-up computed tomography showed good positioning of the internal fixation, and there was no evidence of infection on complete blood count, C-reactive protein, or procalcitonin (PCT) tests. Repeat MRI revealed a large cyst-like hyperintense lesion in the dorsal aspect of the spinal canal from L3 to L5 in the postoperative area, occupying the dorsal space with well-defined margins and signal intensity similar to CSF, suggestive of an epidural pseudocyst (Fig. 2A–C). The lesion significantly compressed the thecal sac, which was displaced anteriorly. The cauda equina nerve roots appeared suspended, clumped, and disorganized, indicating signs of traction. These findings were consistent with postoperative epidural pseudocyst causing cauda equina traction syndrome following laminectomy.

### 2.4. Second surgery and postoperative recovery

**Figure 3.**
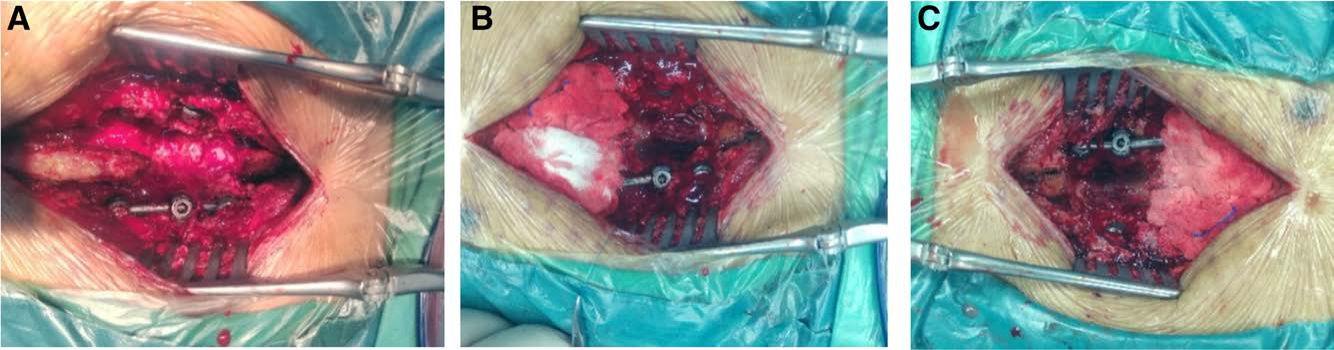
Intraoperative findings and surgical management of cauda equina tethering due to dural defect. (A) Dural rupture with entrapped and swollen cauda equina nerve causing tethering. (B) Careful dissection and release of the entrapped nerve segment extending 0.5 cm bilaterally. (C) Dural repair with continuous 6-0 nerve vascular sutures achieving effective closure.

On June 17, 2022, the patient underwent surgical exploration via the original incision under general anesthesia. After opening the deep fascia and removing a nonabsorbable suture, a thin-walled extradural pseudocyst measuring 5 cm × 3 cm was identified beneath the fascial layer, containing a small amount of colorless, transparent CSF. A defect was observed on the dorsal side of the dura mater, with a portion of the cauda equina entrapped within the defect, resulting in a tethered configuration. The affected cauda equina appeared thickened and swollen due to compression (Fig. 3A), extending approximately 0.5 cm on both ends along the defect. The entrapped cauda equina was carefully freed and released to restore its original compliance (Fig. 3B). The dura mater was sutured using 6-0 nerve vascular sutures, achieving good dural closure (Fig. 3C). After thorough irrigation of the wound with saline solution, the dorsal side of the dura mater was covered with gelatin sponge and sprayed with biological glue. One drain tube was placed at each end of the wound, and the wound was closed layer by layer. On postoperative day 2, the patient’s buttock pain significantly improved, with mild residual numbness in the legs. The drain was removed on day 10, and sutures were removed on day 14. The patient was able to ambulate independently, with only mild residual numbness in the feet, and was discharged on July 14.

At the final follow-up, the patient experienced complete resolution of buttock pain and marked improvement in lower limb sensory deficits. Mild numbness persisted in both feet, but muscle strength and mobility were fully restored. Postoperative MRI performed on July 29, 2022, showed a tension-free membranous cyst (4 cm × 1 cm) behind the decompression site without evidence of recurrent compression. The patient regained independent ambulation and daily self-care abilities and remained symptomatically stable during follow-up.

## 3. Discussion

This case demonstrated that timely surgical exploration and release of the tethered cauda equina nerve resulted in significant symptom relief, functional recovery, and radiological resolution of the compressive pseudocyst. The incidence of postoperative dural tears with CSF leakage is higher after lumbar spine surgery, with most cases being asymptomatic and forming large pseudomeningoceles.^[1,2]^ To prevent delayed wound healing and retrograde bacterial infections such as subarachnoid or intracranial infections due to CSF leakage, it is recommended to tightly suture the drainage site after routine tube removal.^[4]^ Some studies suggest that patients with CSF leakage should avoid early functional exercises. They should instead maintain a supine position with the head low and feet elevated, and apply local compression to the wound to promote dura healing.^[5]^ However, in the current medical environment, the early recovery and early functional exercise concept of enhanced recovery after surgery has been widely promoted.^[6]^ After drainage removal, patients typically begin ambulation with lumbar support. However, in cases of constipation or increased intra-abdominal pressure, elevated intraspinal pressure may cause rupture of the conus medullaris. This can lead to unrelieved local compression, potentially resulting in a tethered cord and symptoms such as lower limb numbness and bowel or bladder dysfunction.^[7,8]^

Unlike most postoperative CSF leaks reported in the literature, which typically present with signs of intracranial hypotension such as postural headache or nausea, or with the formation of pseudomeningoceles due to subcutaneous CSF accumulation,^[9]^ the patient in this case exhibited no classical symptoms of low intracranial pressure. Instead, the predominant presentation was new-onset, progressive bilateral lower extremity pain and numbness, ultimately diagnosed as cauda equina syndrome caused by nerve root traction. In the existing literature, cases of postoperative nerve root adhesion or tethering of the cauda equina are exceedingly rare, and are often attributed to unrecognized or inadequately repaired dural tears, or inappropriate drainage techniques.^[10]^ In this case, no definite dural tear was identified during the initial surgery; however, prolonged negative pressure drainage likely induced a concealed CSF leak, which subsequently led to the formation of an extradural CSF collection and mechanical traction on the nerve roots.

The incidence of cauda equina tethering caused by occult CSF leakage is rarely reported in the literature, likely due to the subtle clinical presentation and difficulties in radiological diagnosis, which may lead to underestimation. Recent case reports and studies suggest that although this complication is rare, it warrants clinical attention.^[11,12]^ High-resolution MRI plays a crucial role in diagnosis, revealing extradural cystic lesions as well as displacement and clustering of the cauda equina nerve roots, indicative of tethering.^[13]^ Management of mild CSF leakage may involve conservative measures such as bed rest and careful wound care, while patients with significant symptoms or neurological deficits require prompt surgical exploration and dural repair.^[12,14–16]^ The core content of the treatment for CSF leakage mainly includes 2 aspects^[17]^: one is to directly repair the hole and block the possibility of CSF leakage; the other is to reduce the pressure in the subarachnoid space or increase the extradural pressure to promote the cessation of CSF leakage.

**Figure 4.**
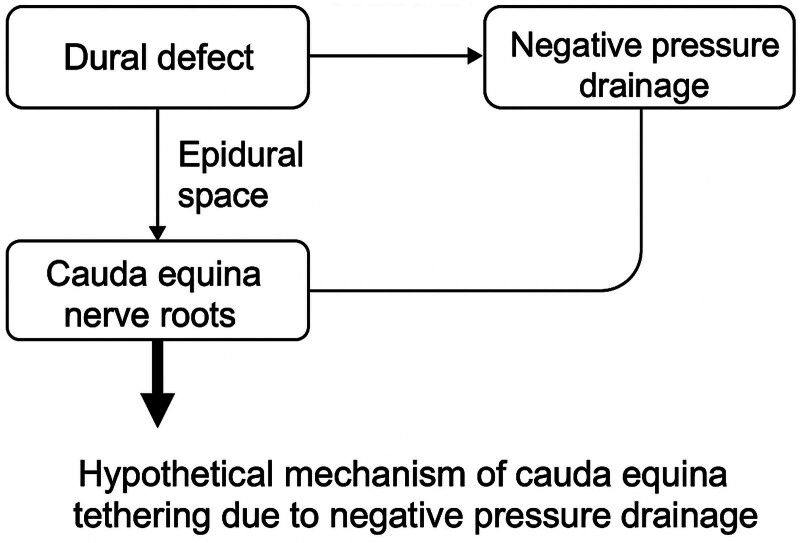
Schematic illustration of the proposed mechanism of cauda equina tethering caused by prolonged negative pressure drainage. Prolonged negative pressure drainage decreases epidural pressure, creating a pressure gradient between the intradural and extradural spaces. CSF leaks through a small dural defect, gradually forming an extradural pseudocyst. This pressure difference and the cystic traction pull a portion of the cauda equina into the dural defect, leading to entrapment and tethering of the nerve roots. CSF = cerebrospinal fluid.

In this case, the existence of CSF leakage has been confirmed through intraoperative and postoperative drainage observation. After the surgery, in order to avoid deep wound fluid accumulation, the placement time of the drainage tube was extended and the drainage tube was removed on the 10th day after the surgery. The use of negative pressure suction drainage may excessively reduce epidural pressure, causing the deep wound pressure to fall below the intradural pressure. This pressure gradient can lead to herniation of the cauda equina through a dural defect. While negative pressure drainage plays a dual role in managing CSF leaks – helping to prevent fluid accumulation and infection – prolonged or excessive suction may generate a traction force that draws nerve roots toward the dural defect, increasing the risk of tethering and herniation (Fig. 4).^[18]^ Alternative strategies include closely monitoring the drainage volume and pressure, limiting the duration of negative pressure application, and considering lumbar CSF drainage to balance the pressure gradient.^[19]^ If neurological symptoms emerge, prompt imaging evaluation and surgical intervention should be considered.

After the first surgical treatment, the patient’s lumbar pain and numbness in the left leg were significantly relieved. The patient’s surgical indication was clear, and the drainage tube was removed and started walking on the 10th day after the surgery without any obstacles. The patient was discharged after the removal of stitches. Severe numbness and pain in the lower limbs occurred on the day after discharge. Firstly, infection was considered, but the patient showed no fever and local redness and swelling of the wound, which did not suggest infection. The reexamination of lumbar spine MRI showed a large extradural pseudocyst after decompression of the spinal canal. Before the second surgery, the author believed that the symptoms were caused by the compression of the dural sac and cauda equina nerves by the cyst, so a second surgical exploration was performed. However, during the surgery, it was found that the tension of the cyst was not significant and there was no obvious fluid accumulation inside the cyst, but the cauda equina nerves were herniated and compressed from the posterior side of the dural sac. On reviewing the postoperative MRI, it was observed that the T2-weighted images showed herniation of part of the cauda equina nerve signals within the dura, and the symptoms were significantly improved after actively reducing the herniated cauda equina nerves during the second surgery. The formation of an epidural pseudocyst may result from CSF leaking into the soft tissue planes, where it becomes encapsulated by fibrous tissue, forming a fluid-filled cavity. This cystic structure can exert compressive effects on the dura mater and adjacent nerve roots.^[20]^ The traction on the cauda equina nerve roots is closely associated with the presence of a dural defect and pseudocyst formation. Entrapped nerve roots may become compressed, swollen, and hypertrophic, as was clearly observed intraoperatively.

As this study presents a single case report, its conclusions have inherent limitations and cannot be generalized to all patients experiencing postoperative CSF leakage. The findings may be subject to reporting bias and lack external validation due to the absence of a control group and long-term follow-up. Future multicenter studies or case series with larger sample sizes are needed to confirm these observations and to further explore optimal management strategies. However, this case highlights the need for heightened clinical vigilance: in patients presenting with persistent or progressive lower limb pain, numbness, or weakness after lumbar surgery – particularly when accompanied by abnormally increased drainage volume – traction injury to spinal nerve roots or the cauda equina should be carefully considered. Postoperative neurological monitoring is essential, and early imaging evaluation or even reoperation may be warranted to promptly identify and manage such rare but serious complications.

Summarily, postoperative occult CSF leakage after lumbar decompression surgery routinely does not require surgical treatment and can heal on its own, with the majority of cases being asymptomatic. After surgery, it is important to avoid secondary infections caused by deep fluid accumulation in the wound, and the use of wound drainage tubes can be extended appropriately. However, once CSF leakage is confirmed, prolonged negative pressure drainage should be avoided to prevent traction of the nerve roots. The duration of drainage must be carefully balanced between reducing the risk of infection and minimizing the potential for neurological compromise. For symptomatic cauda equina tethering syndrome, timely surgical exploration and neurolysis are crucial for relieving nerve compression and improving prognosis.

## Author contributions

**Conceptualization:** Jiaxin Fu, Zhi Liu.

**Data curation:** Chun Zhang, Jiaxin Fu, Shuzhang Guo.

**Formal analysis:** Chun Zhang, Shuzhang Guo.

**Funding acquisition:** Shuzhang Guo, Zhi Liu.
